# Pulmonary Artery Size in Interstitial Lung Disease and Pulmonary Hypertension: Association with Interstitial Lung Disease Severity and Diagnostic Utility

**DOI:** 10.3389/fcvm.2018.00053

**Published:** 2018-06-08

**Authors:** Matthew Chin, Christopher Johns, Benjamin J. Currie, Nicholas Weatherley, Catherine Hill, Charlie Elliot, Smitha Rajaram, Jim M. Wild, Robin Condliffe, Stephen Bianchi, David G. Kiely, Andrew J. Swift

**Affiliations:** ^1^Department of Infection, Immunity and Cardiovascular Disease, University of Sheffield, Royal Hallamshire Hospital, Sheffield, United Kingdom; ^2^Sheffield Teaching Hospitals NHS Foundation Trust, Sheffield, United Kingdom; ^3^Institute for in silico Medicine, Sheffield, United Kingdom

**Keywords:** interstitial lung disease, computed tomography (CT) scanning, right heart catheterisation, pulmonary artery diameter, pulmonary hypertension

## Abstract

**Purpose:**

It is postulated that ILD causes PA dilatation independent of the presence of pulmonary hypertension (PH), so the use of PA size to screen for PH is not recommended. The aims of this study were to investigate the association of PA size with the presence and severity of ILD and to assess the diagnostic accuracy of PA size for detecting PH.

**Methods:**

Incident patients referred to a tertiary PH centre underwent baseline thoracic CT, MRI and right heart catheterisation (RHC). Pulmonary artery diameter was measured on CT pulmonary angiography and pulmonary arterial areas on MRI. A thoracic radiologist scored the severity of ILD on CT from 0 to 4, 0 = absent, 1 = 1–25%, 2 = 26–50%, 3 = 51–75%, and 4 = 76–100% extent of involvement. Receiver operating characteristic analysis and linear regression were employed to assess diagnostic accuracy and independent associations of PA size.

**Results:**

110 had suspected PH due to ILD (age 65 years (SD 13), M:F 37:73) and 379 had suspected PH without ILD (age 64 years (SD 13), M:F 161:218). CT derived main PA diameter was accurate for detection of PH in patients both with and without ILD - AUC 0.873, *p* =< 0.001, and AUC 0.835, *p* =< 0.001, respectively, as was MRI diastolic PA area, AUC 0.897, *p* =< 0.001, and AUC 0.857, *p* =< 0.001, respectively Significant correlations were identified between mean pulmonary arterial pressure (mPAP) and PA diameter in ILD (r = 0.608, *p* < 0.001), and non-ILD cohort (r = 0.426, *p* < 0.001). PA size was independently associated with mPAP (*p* < 0.001) and BSA (*p* = 0.001), but not with forced vital capacity % predicted (*p* = 0.597), Transfer factor of the lungs for carbon monoxide (T_LCO_) % predicted (*p* = 0.321) or the presence of ILD on CT (*p* = 0.905). The severity of ILD was not associated with pulmonary artery dilatation (r = 0.071, *p* = 0.459).

**Conclusions:**

Pulmonary arterial pressure elevation leads to pulmonary arterial dilation, which is not independently influenced by the presence or severity of ILD measured by FVC, T_LCO_, or disease severity on CT. Pulmonary arterial diameter has diagnostic value in patients with or without ILD and suspected PH.

## Introduction

Pulmonary hypertension (PH) is defined on right heart catheterisation (RHC), as a resting mean pulmonary artery pressure (mPAP) greater than or equal to 25 mmHg (1, 2). PH commonly complicates lung disease and chronic hypoxia, such as interstitial lung disease (ILD). When present in lung disease, PH is associated with a poor outcome (3).

CT is used to diagnose and phenotype suspected ILD, and is often part of the workup of patients with unexplained breathlessness and suspected PH (4). Dilatation of the main pulmonary artery (PA) or major branch vessels has been identified as markers of the presence of PH and is often the first imaging finding to suggest the diagnosis (5–9). As CT is commonly used in the investigation of patients with ILD, it would be useful to use the pulmonary arterial size to screen for the presence of pulmonary hypertension. Routine CT pulmonary angiography is performed without ECG gating. Pulmonary arterial size changes during the cardiac cycle. MRI is typically gated to the cardiac cycle and allows assessment of pulmonary arterial size at both systole and diastole. Some authors have suggested that in the presence of established lung fibrosis, the main PA diameter is not accurate for estimation of mean pulmonary arterial pressure as dilatation of the main PA develops in patients with pulmonary fibrosis in the absence of PH (5, 10).

The aim of this study was to investigate the role of main pulmonary arterial size in patients with ILD and suspected pulmonary hypertension. Firstly, by investigating the factors associated with main pulmonary arterial dilatation, including markers of disease severity in interstitial lung disease. Secondly, to compare the diagnostic accuracy of PA size in patients with suspected pulmonary hypertension with and without ILD.

## Methods

### Patients

Consecutive patients who were referred to a pulmonary hypertension centre from 24 April 2012 to 30 March 2016 were identified from the ASPIRE registry (3). Patients with a CT scan within 90 days of MRI and RHC were included. In order to meet inclusion criteria, a diagnostic quality CT pulmonary angiogram (CTPA) with a slice thickness of less than 5 mm was required. Patients underwent systemic evaluation as part of their routine clinical workup, which included clinical review, multi-modality imaging and lung function testing.

The aetiology of pulmonary hypertension was decided at a multi-disciplinary team meeting, based upon review of radiological, RHC and clinical information. The North Sheffield Ethics Committee approved this study and institutional review board approval was attained.

All patients with ILD were assessed for radiological disease pattern on CT. The most common pathological patterns of fibrosis referred to our PH centre are usual interstitial pneumonia (UIP) and non-specific interstitial pneumonia (NSIP). These groups were prospectively separated to examine for differences between these groups.

### CT Acquisition

The majority of CTPA scans (76.8%) were conducted at Sheffield’s Pulmonary Vascular Disease Unit with a further 114 cases performed at the referring hospitals in Wales or the North of England. All Sheffield CTPA cases were performed on a 64-slice MDCT scanner (light-speed General Electric Medical Systems, Milwaukee, WI), with standard acquisition parameters: 100 mA with automated dose reduction, 120 kV, pitch 1, rotation time 0.5 s and 0.625 mm slice thickness. A 400 × 400 mm field of view was used with an acquisition matrix of 512 × 512. 100 ml of intravenous contrast agent (Ultravist, Bayer, Berlin, Germany) was administered at a rate of 5 ml/sec through a wide bore cannula, into a large central vein, typically in the ante-cubital fossa. The scan was then timed using a bolus tracking technique. HRCT was reconstructed using the contrast-enhanced acquisitions with 1.25 mm collimation from the apex of the lung to the diaphragm. In order to be included, any CTPA from an outside Trust had to be of diagnostic quality, as decided by a radiologist and slice thickness ≤5 mm. In the 23.2% (114) CTPAs performed outside of Sheffield, CT scanner and detailed acquisition parameters were not available for reporting.

### MR Acquisition

Cardiac magnetic resonance (CMR) imaging was performed on a 1.5T whole body scanner GE HDx (GE Healthcare, Milwaukee, USA), using an 8-channel cardiac coil. Patients were in the supine position with a surface coil and with retrospective ECG gating. A retrospective cardiac gated multi-slice balanced steady state free precession (bSSFP) sequence was performed orthogonal to the main pulmonary artery. The bSSFP sequence parameters were: TR 2.8 ms, TE 1.0 ms, Flip angle of 50°, FOV = 48 × 43.2, 256 × 256 matrix, 125 kHz bandwidth and slice thickness of 10 mm (11).

### Pulmonary Function Testing

All patients underwent lung function testing. Percent of predicted values for forced vital capacity (FVC), and transfer factor of the lungs for carbon monoxide (T_LCO_) were calculated. The gender, age and physiology (GAP) score is a simple tool for estimating mortality in ILD from demographic and pulmonary function metrics and was calculated as previously described (12).

### CT Image Analysis

Image analysis was carried out on picture archive and communication system (PACS) imaging on CE stamped Barco diagnostic monitors. The observer was blinded to the all other clinical and imaging data.

**Figure 1:**
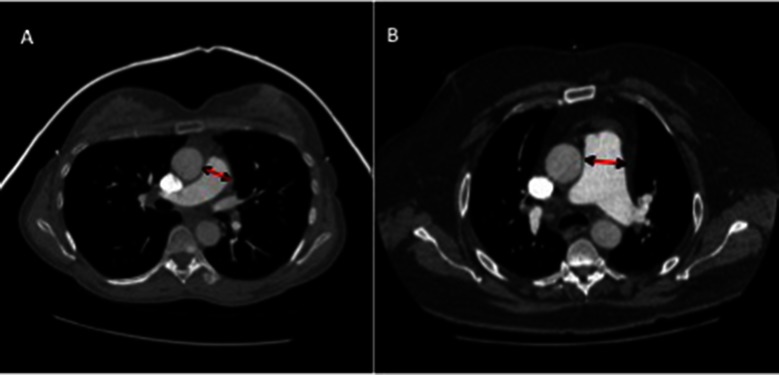
CT images of pulmonary artery diameter measurements in patients with **(A)** a patient without PH (mean pulmonary arterial pressure of 20 mmHg), **(B)** a patient with PH with moderate elevation in pulmonary arterial pressure (mPAP 54 mmHg). Diameter measured where largest and most consistent - proximal to bifurcation, perpendicular to direction of vessel.

The main pulmonary artery was measured proximal to bifurcation, perpendicular to the direction of the vessel at the point where largest diameter is most consistent. The ascending aorta diameter was also measured on the same CT slice, see Figure 1. Right and left pulmonary artery diameters were recorded 1 cm from the bifurcation, at their most consistent value. A thoracic radiologist scored the severity of ILD on CT, using the Likert semi-quantitative score, ranging from 0 to 4, 0 = absent, 1 = 1–25%, 2 = 26–50%, 3 = 51–75%, and 4 = 76–100% involvement (13). The imaging subtype of ILD was also recorded (UIP or NSIP).

### MR Image Analysis

Image analysis was performed on a GE Advantage Workstation 4.4 and GE Advantage Workstation Report Card. Scans were defined as non-diagnostic when image quality significantly affected cardiac measurements or volumetric analysis could not be accurately performed. From the magnitude phase imaging images, the maximal (systolic) and minimal (diastolic) PA areas were measured, and relative area change (RAC) was defined by the following equation: RAC = (maximum area-minimum area)/minimum area (14, 15).

### Statistics

The differences in CT and MRI pulmonary arterial size between the patients with and without ILD were analysed using an independent *t*-test for continuous data and the chi-square for categorical data. ANOVA with Bonferroni correction was used for multiple group comparison. Pearson’s correlation coefficient was used to assess the correlation between CT and MRI pulmonary arterial measurements and mPAP. Pearson correlations were conducted for cohorts of ILD, non-ILD and radiological subtypes against CT and MRI derived metric variables. Receiver operating characteristic (ROC) analysis was performed to determine diagnostic accuracy of CT and MRI pulmonary arterial size measurements with area under the ROC curve (AUC) results presented. Multiple t-tests were used to assess intergroup variance of PA diameter, PA to Aorta (PA:AA) ratio and PA relative to body mass index (PA index). The relationship between PA diameter with candidate predictors mPAP, ILD severity of CT, FVC T_LCO_, and age, sex and body surface area (BSA) was assessed using multivariate linear regression analysis.

Statistical analysis was performed in IBM SPSS Statistics 22 (SPSS, Chicago) and graphed in GraphPad Prism (GraphPad, San Diego). A two-tailed *p*-value of <0.05 was considered statically significant.

## Results

### Patients

**Figure 2:**
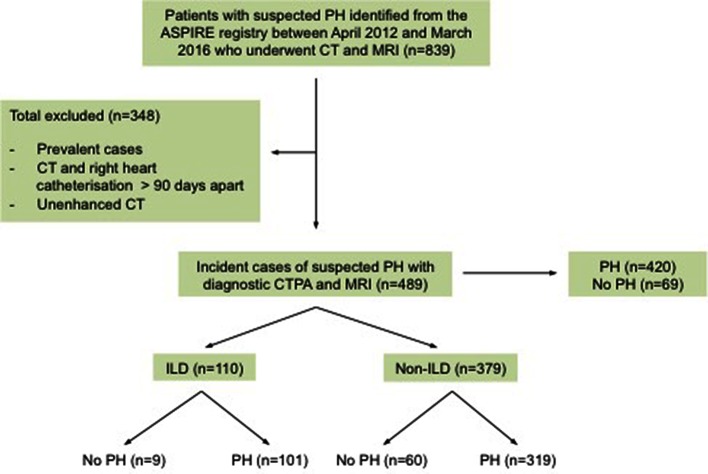
The Study Cohort. PH, pulmonary hypertension; CT, computed tomography scan; MRI, magnetic resonance imaging; MPA, main pulmonary artery; CTPA, computed tomography pulmonary angiogram; ILD, interstitial.

From the ASPIRE database 489 patients with suspected PH were identified, including 198 males and 291 females. 420 patients had pulmonary hypertension. The mean age for the whole cohort was 65 (SD - SD 13). One hundred and ten patients had CT features of ILD (101 patients with pulmonary hypertension and 9 patents without), of which 46 patients had UIP and 43 had NSIP radiological subtypes. Chronic EAA (*n* = 1), asbestosis (*n* = 1), desquamative interstitial pneumonia (*n* = 1), chronic extrinsic allergic, alveolitis (*n* = 1), silicosis (*n* = 1), Langerhans cell histiocytosis (*n* = 1), radiotherapy (*n* = 1) and post infective interstitial disease and scarring (*n* = 4). 379 patients with suspected PH had no CT evidence of ILD (319 patients with pulmonary hypertension and 60 patients without) as shown in Figure 2**. **Table 1 provides the baseline demographic data, lung function, CT and MRI derived variables for the study cohort as a whole and split into the ILD and not ILD cohorts. The mean (SD) interval between CT and RHC was 17.6 days (46.1), the interval between MRI and RHC was 0.2 days (4.2).

**Table 1:** Demographic table.

		**Whole Cohort**	**ILD**	**Non-ILD**	*p* Value*
		**Mean (SD)* N* = 489**	**Mean (SD) *N* = 110**	**Mean (SD) *N* = 379**	
**Demographics**					
**Age**		65 (13)	66 (11)	64 (13)	0.656
**Sex**	M/F	198/291	37/73	161/218	
	PH/No PH	420/69	101/9	319/60	0.003
**WHO Functional Class**	I/II/III/IV	1/59/378/42	0/9/80/18	1/50/298/24	<0.001
**ILD Sub-Types**	UIP/NSIP/Sarcoid/Chronic EAA, Other		46/43/11/1/20		
**Right Heart Catheter**					
	mRAP (mmHg)	10 (6)	9 (6)	10 (5)	0.017
	mPAP (mmHg)	41 (15)	41 (13)	42 (15)	0.856
	PAWP (mmHg)	13 (5)	12 (5)	13 (5)	0.014
	CI (L/min/m2)	2.7 (0.8)	2.7 (0.8)	2.7 (0.8)	0.981
	PVR (dyn.s.m-5)	529.2 (394.3)	539.7 (348.7)	526.1 (408.1)	0.267
**Pulmonary function tests**					
	Predicted FVC %	81.6	72.7	85.0	<0.001
	FVC (l)	2.9 (1.1)	2.4 (0.9)	3.1 (1.1)	<0.001
	Predicted T_LCO_ %	47.9	33.7	52.3	<0.001
	T_LCO_ (l)	3.9 (2.0)	2.5 (1.6)	4.3 (2.0)	<0.001
**CT**					
	Main PA diameter (mm)	32 (6)	31 (4.9)	32 (5.8)	0.092
	PA/AA	1 (0.2)	1 (0.2)	1 (0.2)	0.453
	PA/BSA	17.6 (3.5)	18 (3.0)	18 (3.6)	0.434
	Right PA diameter (mm)	26 (5)	25 (4.4)	26 (4.7)	0.234
	Left PA diameter (mm)	24 (4)	24 (3.4)	25 (4.0)	0.309
**MRI**					
	Systolic PA area (mm^2^)	920 (306)	887 (269)	930 (316)	0.607
	Diastolic PA area (mm^2^)	833 (276)	807 (255)	814 (281)	0.809
	PA relative area (mm^2^)	10.9 (9.5)	10.6 (7.2)	10.9 (9.3)	0.394

WHO, World Health Organisation; mRAP, mean Right Arterial Pressure; mPAP, mean Pulmonary Arterial Pressure; PAWP, Pulmonary Artery Wedge Pressure; CI, Cardiac Index; PVR, Pulmonary Vascular Resistance; TLCO, Transfer Factor of the lungs for Carbon Monoxide; FVC, Forced Volume Capacity.

### Group Comparisons

**Figure 3:**
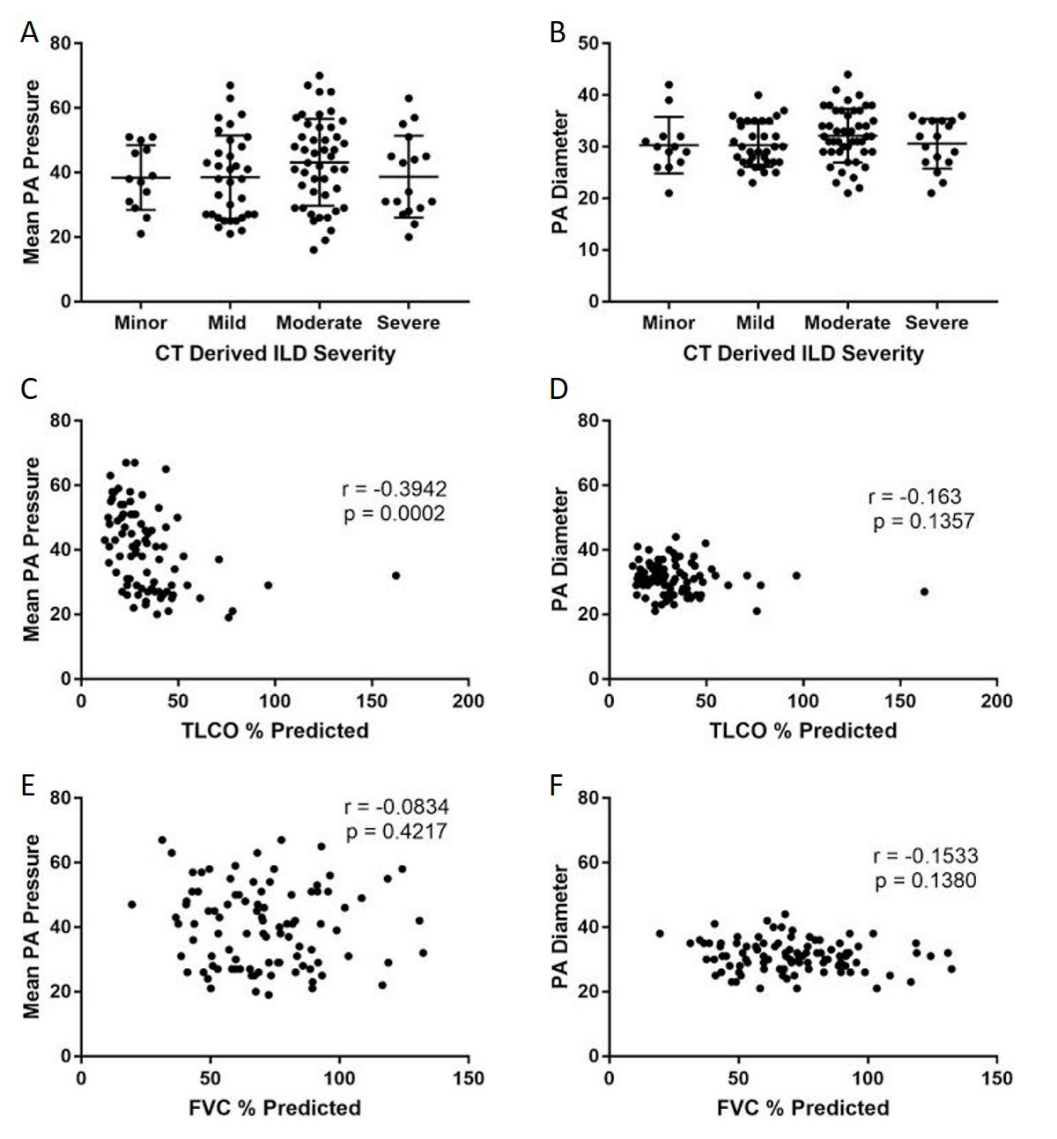
Correlations between mean PA pressure and PA diameter with pulmonary function tests and CT-derived severity score. Graphs **(A)** and **(B)** represent the association with ILD severity on CT in ILD cohort. Graphs **(C)** and **(D)** scatter plot showing the association of PA pressure and diameter with T_LCO_ %predicted and graphs **(E)** and **(F)** scatter plots showing the association of PA pressure and diameter with FVC %predicted.

Patients with PH had larger pulmonary arteries (33 mm) than patients without PH (27 mm) (*p* < 0.001) In the ILD cohort, there was no significance between PA size diameters in different semi-quantitative radiological severity scores of ILD, see (Figure 3A, mPAP and ILD severity). There was no difference in mPAP between radiological severities of ILD scores (Figure 3B, PA diameter and ILD severity). Furthermore there were no significant differences in PA size or diameter between CT derived ILD severity within the pathological subgroups, UIP *p* = 0.630 and NSIP *p* = 1.000.

### Correlations

**Figure 4:**
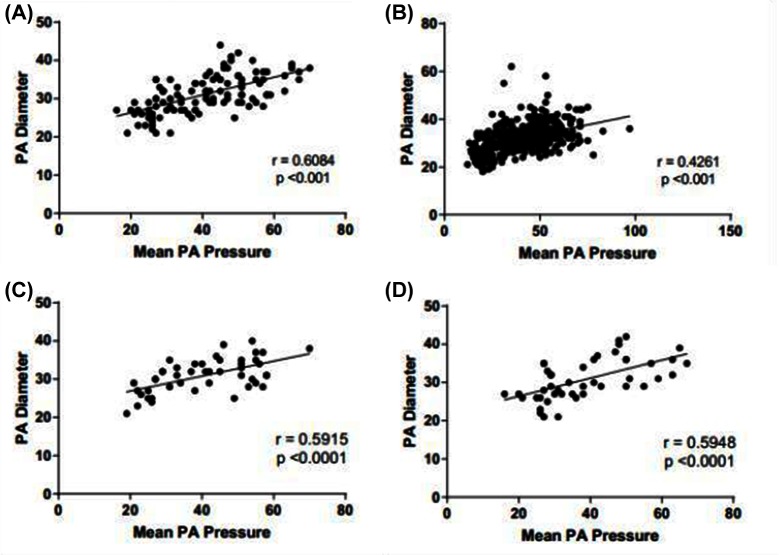
Scatter distribution showing the relationship between mean PA pressure and PA diameter. Subgroups include ILD cohort **(A)**, non-ILD **(B)**, ILD with UIP pattern of disease **(C)** and ILD with NSIP pattern of disease **(D)**.

CT derived main PA diameter correlated positively with mPAP in the ILD cohort (r = 0.608; *p* < 0.001), which improved when the pulmonary artery diameter was indexed to body surface area (r = 0.674 ; *p* < 0.001). MRI derived systolic and diastolic pulmonary artery area also correlated significantly with mPAP in the ILD cohort, (r = 0.423 and 0.479 respectively, *p* < 0.001). In the pathological subtypes, PA diameter correlated with mPAP for both UIP (r = 0.592; *p* < 0.001) and NSIP (r = 0.621; *p* < 0.001). Each are shown in Figure 4**.** In the non-ILD cohort, the correlation between mPAP and PA diameter was weaker but still significant, r = 0.426 (*p* < 0.001). Table 2 presents Pearson correlations for ILD and non-ILD cohorts, with pathological UIP and NSIP subtype analysis. In the ILD cohort, there was no significant correlation between PA diameter and FVC (r = −0.113; *p* = 0.301) or T_LCO_ (r = −0.041; *p* = 0.692) respectively, as shown in Figure 3 shows the correlations between mean PA pressure and PA diameter for ILD (r = 0.608; *p* < 0.001), non-ILD (r = 0.461; *p* < 0.001), UIP (r = 0.592; *p* < 0.001), NSIP (r = 0.595; *p* < 0.001).

**Table 2:** Correlations of CT and MRI measurements with mean pulmonary arterial pressure.

**Pearson Correlations to mPAP**	**ILD**		**UIP**		**NSIP**		**Non ILD**	
	*R Value*	*p Value*	*R Value*	*p Value*	*R Value*	*p Value*	*R Value*	*p Value*
**CT**								
PA Diameter	0.608	<0.001	0.592	<0.001	0.621	<0.001	0.426	<0.001
PA/AA Ratio	0.478	<0.001	0.515	<0.001	0.631	<0.001	0.431	<0.001
PA Index	0.498	<0.001	0.374	0.01	0.674	<0.001	0.402	<0.001
Right PA Diameter	0.466	<0.001	0.396	0.006	0.472	0.002	0.262	<0.001
Left PA Diameter	0.393	<0.001	0.406	0.005	0.656	0.029	0.332	<0.001
**MRI**								
Systolic PA area	0.423	<0.001	0.461	0.001	0.510	<0.001	0.353	<0.001
Diastolic PA area	0.479	<0.001	0.563	<0.001	0.558	<0.001	0.426	<0.001
PA relative areachange	−0.397	<0.001	−0.457	0.001	−0.387	0.010	−0.310	<0.001

AA , Ascending Aorta; ILD , Interstitial Lung Disease; mPAP , mean Pulmonary Artery Pressure; NSIP , Non-specific Interstitial Pneumonia; PA , Pulmonary Artery; UIP , Usual Interstitial Pneumonia.

### Diagnostic Accuracy

Table 3 provides the ROC area under the curve (AUC) values for CT and MRI metrics in both ILD and non-ILD cohorts for the diagnosis of PH. In the ILD cohort, main PA diameter on CT had strong diagnostic accuracy, AUC = 0.873 as did systolic and diastolic PA area on MRI (AUC = 0.887 and AUC = 0.897, respectively). In addition, high diagnostic accuracy of PA diameter on CT, AUC 0.835 and PA systolic and diastolic areas (AUC 0.824 and 0.857, respectively) on MRI were found in the non-ILD cohort. The relative area change of the pulmonary artery during the cardiac cycle was a weaker diagnostic marker than pulmonary arterial size.

**Table 3:** Area under the receiver operating characteristic curve for CT and MRI pulmonary arterial measurements in ILD and non-ILD cohorts.

**CT and MRI Metrics**	**ILD**		**Non ILD**	
	AUC	*p* value	AUC	*p* value
**CT**				
Main PA Diameter	0.873	<0.001	0.835	<0.001
PA/AA	0.799	0.003	0.794	<0.001
PA Index	0.595	0.347	0.836	<0.001
Right PA Diameter	0.753	0.012	0.757	<0.001
Left PA Diameter	0.729	0.023	0.754	<0.001
**MRI**				
Systolic PA area	0.887	<0.001	0.824	<0.001
Diastolic PA area	0.897	<0.001	0.857	<0.001
PA relative area change	0.388	0.266	0.365	<0.001

AA , Ascending Aorta; PA , Pulmonary Artery.

**Figure 5:**
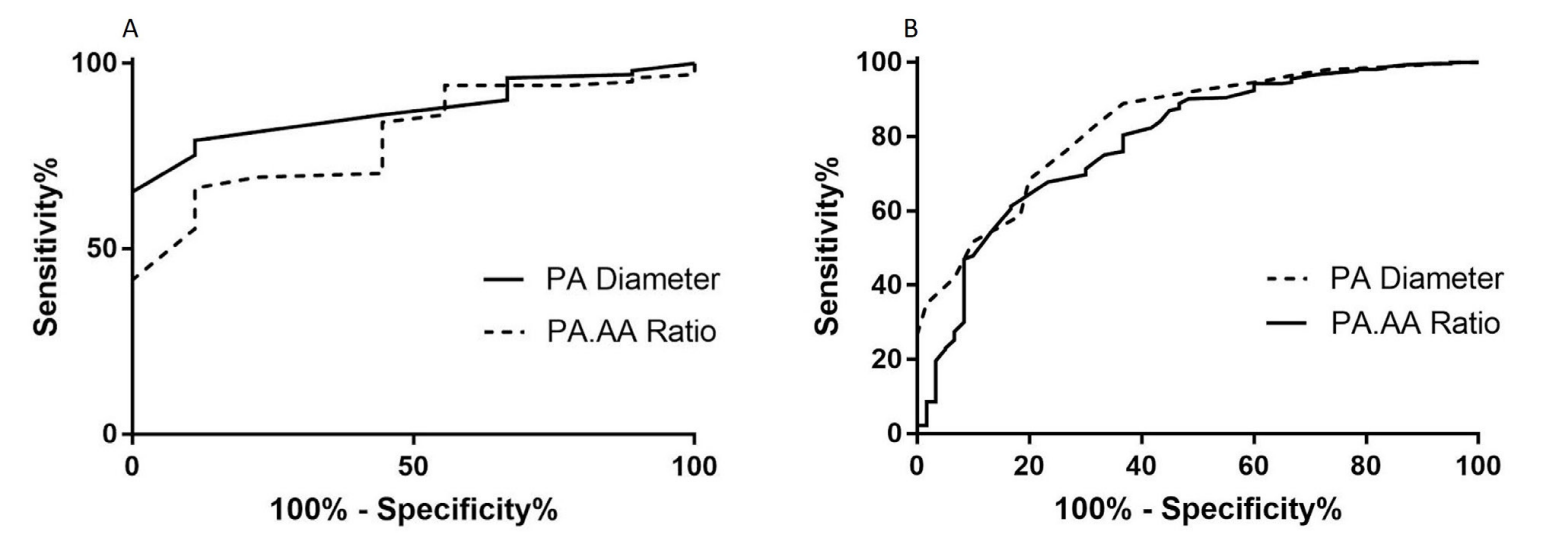
ROC analysis showing the area under the curve for both ILD **(A)** and non-ILD **(B)** cohorts for the CT measured main pulmonary artery size (PA diameter and PA:AA Ratio)

A previous threshold of 29mm tested in the ILD cohort resulted in 75.2% sensitivity, 88.9% specificity, 98.7% positive predictive values and 24.0% negative predictive value. In the non-ILD cohort, pulmonary arterial diameter was also accurate for the diagnosis of PH, with AUC=0.835 (*p*<0.001). The optimal threshold identified was 30mm, providing 76.3% sensitivity and 73.3% specificity. Figure 5 shows the ROC curves for the CTPA measured pulmonary arterial diameter metrics (PA diameter, PA:AA ratio and PA index) in both ILD and non-ILD cohorts.

The highest AUC values for MRI derived variables in the ILD cohort (Table 2) were diastolic pulmonary arterial area (0.897) and systolic pulmonary arterial area (0.887). MRI variables have shown a similar diagnostic accuracy within this study cohort compared to CT derived parameters of PA size.

### Factors Associated with Pulmonary Arterial Size in ILD

At multivariate linear regression analysis, mPAP and body surface area were independent predictors of pulmonary arterial size, [11.1 + (0.2 × mean pulmonary arterial pressure) + (6.7 × BSA)]. Age, sex, GAP score, ILD severity on CT, FVC %predicted and T_LCO_ %predicted were not multivariate predictors of pulmonary artery diameter (10). Table 4 shows linear regression analysis. Mean pulmonary arterial diameter had an adjusted correlation coefficient of 0.575 (*p* < 0.001) and BSA of 0.249 (*p* = 0.001).

**Table 4:** Linear regression analysis, identifying covariates with independent association with pulmonary arterial diameter.

**Model**	**Regression Co-efficient**	SE	**R**	**T Statistic**	***p* value**
(Constant)	11.060	3.471		3.187	0.002
PA Mean	0.205	0.033	0.525	6.168	<0.001
BSA	6.738	1.914	0.300	3.520	0.001

BSA, Body Surface Area; PA, Pulmonary Artery.

## Discussion

In this retrospective study of 491 patients with suspected pulmonary hypertension referred to a tertiary referral centre, we show that main pulmonary arterial diameter has similar diagnostic utility in patients with and without ILD. In our cohort, pulmonary arterial diameter is independently associated with mean pulmonary arterial pressure and body size. The size of the pulmonary artery was not independently influenced by semi-quantitative radiological severity score of ILD, FVC, T_LCO_, or GAP score.

It has been postulated that in patients with pulmonary fibrosis the pulmonary artery dilates in the absence of elevated pulmonary arterial pressure, as consequence of architectural distortion of the lung parenchyma. In contrast, in this cohort of patients, we have found that pulmonary arterial diameter is diagnostic of pulmonary hypertension in ILD, to a similar accuracy as seen in patients with pulmonary hypertension who do not have ILD (AUC 0.874 for ILD cohort and AUC 0.835 for non-ILD cohort), irrespective of the underlying radiological ILD pattern. UIP is typified by significant parenchymal distortion, including honeycombing, traction bronchiectasis and volume loss (16), therefore if PA size is driven by architectural destruction in ILD, it should be apparent in this group. However, PA size remains more closely associated with PH metrics than quantitative metrics of ILD severity in patients with UIP. In addition, the optimal pulmonary arterial diameter threshold for prediction of elevated mean pulmonary artery pressure was relatively low in patients with ILD at 29 mm, similar to 30 mm for patients in the non-ILD cohort. Mean pulmonary arterial diameter in our ILD cohort with pulmonary hypertension was 32 mm, compared to a mean of 25 mm in patients with ILD without pulmonary hypertension.

As expected, T_LCO_ and FVC were lower in the patients with ILD than those without. However, the ILD and non-ILD cohorts were well-matched in terms of PH severity, as defined by their RHC metrics. The clinical and radiological markers of ILD severity (T_LCO,_ FVC and CT semi-quantitative severity score) had weak correlations with pulmonary arterial diameter (r = −0.148, *p* = 0.174/r = −0.153, *p* = 0.138/r =0.082, *p* = 0.393 respectively). Furthermore, there is a strong correlation between PA diameter and mean PA pressure within the ILD cohort (r = 0.608). On multivariate linear regression, the clinical and radiological markers of disease severity were not independent predictors of pulmonary arterial size. In fact, the independent predictors were body surface area and pulmonary arterial pressure.

Both CT and MRI derived pulmonary arterial size metrics were assessed. MRI variables showed high diagnostic accuracy with both systolic and diastolic pulmonary arterial area having diagnostic utility in both ILD and non-ILD cohorts. MRI derived diastolic pulmonary arterial area, was the most accurate diagnostic variable across the whole study cohort, regardless of the presence of ILD. Diastolic pulmonary arterial size was of systematically marginally higher diagnostic accuracy than systolic. We postulate that this is because of greater pulsatility of the pulmonary artery of a patient without pulmonary hypertension, allowing the pulmonary artery to reduce in size in diastole to a greater extent relative to a patient with pulmonary hypertension.

PA size measured on MRI also correlated well with RHC derived mean pulmonary arterial pressure and showed similar diagnostic accuracy to CT derived PA diameter. This trend was similar in both patients with and without interstitial lung disease. A limitation of routine CT pulmonary angiography is lack of cardiac gating; MRI with cardiac gating has provided additional proof of the equivalent utility of PA diameter in interstitial lung disease and non- interstitial lung disease

The pulmonary artery can dilate over time in a patient with elevated pulmonary arterial pressure (17). Hence the absolute instantaneous measure of pulmonary arterial pressure in a patient with pulmonary hypertension will be dependent not only on the pressure but also the duration of the disease, which explain the variable correlation observed between pulmonary arterial size and pressure in patients with suspected PH (5–7, 11, 18–20). All patients were referred with suspected pulmonary hypertension, however it is unknown how long the pressure has been elevated, given the insidious onset of the disease, absolute quantification of duration of disease is challenging.

## Limitations

This study is limited by its retrospective design at a single tertiary referral centre, although in order to reduce bias from the retrospective analysis, the cohort is made up of consecutive patients. As this was performed in a pulmonary hypertension referral centre, there is a bias towards the presence of PH and as such there are only a few patients with no PH, so these results are only valid in the setting of a PH referral centre. It is expected, however that the strong correlation between mPAP and PA size in this cohort of patients would be similar in a non-selected patient cohort. A study in a non-PH centre, in a cohort with more even distribution of patients with and without pulmonary hypertension would be of benefit.

Interstitial lung diseases are a heterogeneous group of diseases with idiopathic and known-cause aetiologies. We have grouped the largest cohorts of fibrotic ILDs here to explore if the architectural destruction of UIP had a greater influence on PA dilatation than NSIP, but in practice these are not clinical diagnoses. However, in practice these are the patterns most likely to lead to fibrotic-driven architectural distortion and so we have taken a pragmatic approach in separating these. Further work evaluating the impact of varying forms of ILD, such as scleroderma related and drug induced ILD on the pulmonary vasculature, in terms of PA size and haemodynamic changes would be of value.

## Conclusion

Pulmonary arterial pressure elevation leads to pulmonary arterial dilation, which is not independently influenced by the presence or severity of ILD measured by FVC, T_LCO_, or disease severity on CT. Pulmonary arterial diameter has diagnostic value in patients with or without ILD, key for the screening of suspected PH.

## Ethics Statement

This study was carried out in accordance with the recommendations of “North Sheffield Ethics Committee”. The protocol was approved by the “Sheffield Hospitals institutional review board”.

## Author Contributions

AS and DK conceived the idea for the study. AS, MC, NW, SB, BC and CJ participated in the study design. AS, DC acquired the MRI data. Image analysis was performed by AS, MC, BC, CJ. AS, MC, SR, CE, CJ, RC, DK, NW, JMW analysed and interpreted the MR data. AS, MC, SR, CE, CJ, NW, RC, DK, JMW, SB drafted the manuscript. All authors read and approved the final manuscript.

## Conflict of Interest Statement

The authors declare that the research was conducted in the absence of any commercial or financial relationships that could be construed as a potential conflict of interest.

## Abbreviations

AA: Ascending Aorta
ILD: Interstitial Lung Disease
mPAP: mean Pulmonary Artery Pressure
NSIP: Non-specific Interstitial Pneumonia
PA: Pulmonary Artery
UIP: Usual Interstitial Pneumonia
